# Clinical characteristics of venous thromboembolism onset from severe high altitude pulmonary edema in plateau regions

**DOI:** 10.1186/s12959-023-00469-4

**Published:** 2023-02-28

**Authors:** Yanmin Liu, Xinwei Feng, Yongxue Tang, Yanqiu Sun, Xiaoyan Pu, Xiaokai Feng

**Affiliations:** 1 Department of Cardiology, Qinghai Provincial People’s Hospital, 2 Gonghe Road, Chengdong District, Xining, Qinghai Province 810007 China; 2 Suzhou Medical College of Soochow University, 199 RenAi Road, Suzhou Industrial Park, Suzhou, Jiangsu Province 215123 China; 3 College of Medicine, Qinghai University, 16 Kunlun Road, Chengxi District, Xining, Qinghai Province 810001 China; 4 The Department of Radiology, Qinghai Provincial People’s Hospital, 2 Gonghe Road, Chengdong District, Xining, Qinghai Province 810007 China; 5 Department of Respiratory and Critical Care Medicine, Qinghai Provincial People’s Hospital, 2 Gonghe Road, Chengdong District, Xining, Qinghai Province 810007 China; 6 Department of Respiratory and Critical Care Medicine, Beijing Institute of Respiratory Medicine and Beijing Chao-Yang Hospital, Capital Medical University, No. 8, Gongti South Road, Chaoyang District, Beijing, 100020 China

**Keywords:** High altitude pulmonary edema, Venous thromboembolism, High altitudes, Clinical characteristics, Thromboprophylaxis

## Abstract

**Background:**

To investigate venous thromboembolism (VTE) in hospitalized patients with severe high altitude pulmonary edema (HAPE), we performed a single center retrospective study to evaluate its clinical characteristics, prognosis, and potential thromboprophylaxis strategies in a large referral and treatment center in plateau regions.

**Methods:**

We studied a total of 18 patients with severe HAPE from January 1, 2012 to December 31, 2021. Demographic and clinical data, laboratory data, including ultrasound scans of the lower extremities and cardiac ultrasound, and computed tomographic pulmonary angiography (CTPA) variables were obtained, and comparisons were made between groups with and without VTE.

**Results:**

Of the 18 patients hospitalized with severe HAPE (age 43 (range, 34–54) years, 14 [77.8%] men), 7 patients developed VTE (38.9%), including 5 with deep vein thrombosis (DVT) and pulmonary embolism (PE), 2 of whom had DVT only. Eighteen patients are all firstly rapid ascent to high altitudes which the mean altitude was 3700 m (3656–4050 m). Compared with patients who did not have VTE, patients with VTE had a longer time in hospital (13 [11, 19] versus 9 [7, 12]; *P* = 0.027), respiratory failure (6 [85.7%] versus 2 [18.2%]; *P* = 0.013), the shortened APTT (21.50 [19.00, 27.50] versus 26.30 [24.80, 30.10]; *P* = 0.044) and the higher level of D-dimer (7.81 [4.62, 9.60] versus 2.90 [1.75, 3.37]; *P* = 0.003). The proportion of thromboprophylaxis is too low in our cohort which 2 of 18 (11.1%) patients were given VTE prophylaxis. There was no statistically significant difference between the VTE and non-VTE groups (0 [0.0%] versus 2 [18.2%]; *P* = 0.497).

**Conclusions:**

The prevalence of VTE is high in hospitalized patients with severe high altitude pulmonary edema (HAPE). Prophylaxis for venous thromboembolism may be protective in severe HAPE patients after admission. Our data seem to suggest that VTE is probably an additional prognostic factors in patients with severe HAPE.

## Introduction

High altitude pulmonary edema (HAPE) is a series of pulmonary disorders caused by pulmonary vasoconstriction due to hypoxia when a person first enters a plateau (usually > 2500 m above sea level). HAPE is the most common cause of death related to high altitude. The reported incidence of HAPE ranges from an estimated 0.01% of skiers traveling from low altitude to Vail, CO (2500 m), to 15.5% of Indian soldiers rapidly transported to altitudes of 3355 and 5940 m [1]. It is a non-cardiogenic pulmonary edema and a potentially fatal disease of altitude. Exaggerated hypoxic pulmonary vasoconstriction, elevated pulmonary artery pressures, and high-permeability noncardiogenic edema resulting from stress failure of pulmonary capillaries in focal areas of the lung characterize HAPE. Early symptoms of HAPE include exertional dyspnea, cough and reduced mobility. As the disease progresses, dyspnea at rest, worsening cough and the appearance of distinct pink frothy sputum suggest the development of significant pulmonary edema [2]. Deep vein thrombosis (DVT), a subset of venous thromboembolism (VTE), is a major preventable cause of morbidity and mortality worldwide. The incidence of VTE is estimated to be 1 per 1000 people annually [3, 4]. Virchow’s Triad, first described in 1856, implicates three contributing factors in the formation of thrombosis: venous stasis, vascular injury, and hypercoagulability. However, the concurrent presence of venous stasis and vascular injury or hypercoagulability greatly increases the risk for clot formation [5].

Previous studies demonstrated an increased risk of venous stasis, vascular injury, and hypercoagulability in HAPE patients. The prevalence of VTE and its pathophysiology, clinical characteristics, prognosis, screening, and preventive strategies have not been investigated in this HAPE illness that can cause severe and critical disease and death. We performed a single institutional retrospective study in patients with confirmed severe HAPE to identify the prevalence, clinical characteristics, and prognosis of VTE in the cohort of hospitalized patients.

## Methods

### Study design and patients

Two hundred and sixty-four patients diagnosed with HAPE at Qinghai Provincial People’s Hospital between January 1, 2012 and December 31, 2021 were retrospectively collected.

**Fig. 1:**
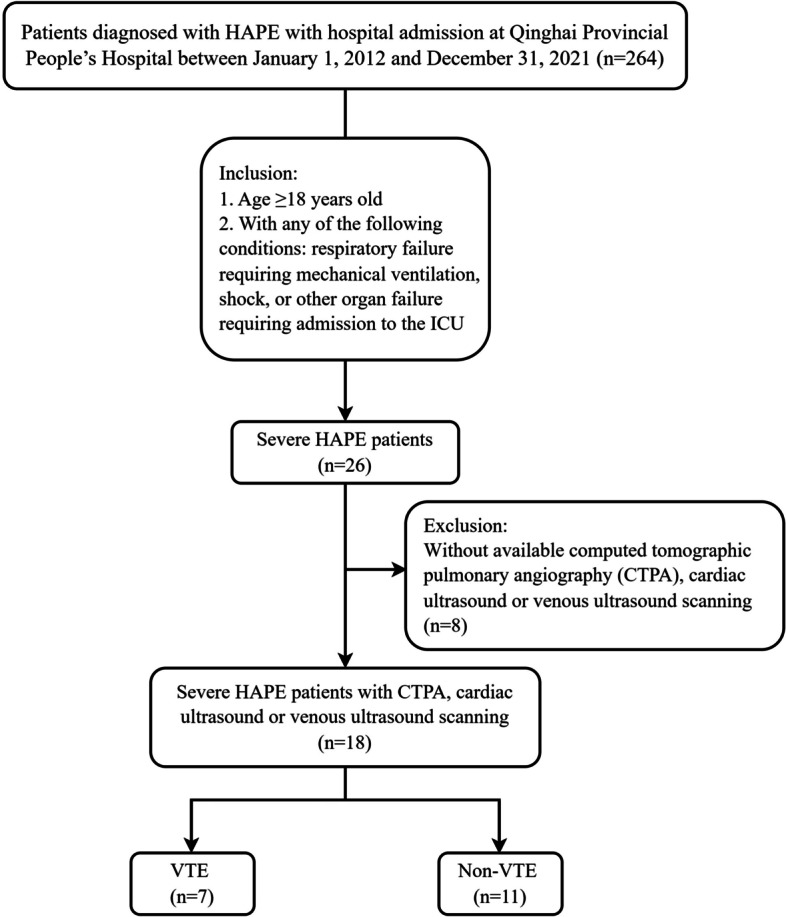
Flow chart of the study

The inclusion criteria were 1) diagnosed with HAPE according to the Guideline [6]; 2) aged ≥18 years; 3) any of the following conditions: respiratory failure requiring mechanical ventilation, shock, or other organ failure requiring admission to intensive care unit (ICU) admissions. Therefore, 26 patients with severe HAPE were included. Then, we excluded those without available computed tomographic pulmonary angiography (CTPA), cardiac ultrasound or venous ultrasound scanning. We divided the above population into VTE and non-VTE groups in order to effectively explore the clinical characteristics of VTE. Finally,18 patients with severe HAPE were included in the analysis (Fig. 1).

### Data collection

The baseline characteristics of the patients were collected from the medical records, including age, sex, weight, height, smoking history, altitude (permanent residence, birthplace and affected area), altitude difference, disease course, hospital stay, medical history and complications. Body mass index (BMI) was calculated as weight (kg) divided height squared (m^2^). The symptoms, physical examination findings, laboratory examination findings at admission, echocardiographic detection and treatments were collected.

### Statistical analysis

The normality of the continuous variables was tested using the Shapiro-Wilk test. The data in a normal distribution were described as means ± standard deviations and compared using the independent students’ *t*-test. The data not in a normal distribution were described as medians (P25, P75) and compared using the nonparametric Wilcoxon test. The categorical data were described as n (%) and compared using the Fisher exact probability test. We used two-tailed test, and *P* < 0.05 was considered statistically significant. All statistical analyses were performed using SAS 9.4 (SAS Institute, Cary, NC, USA) and R (version 3.6.3, https://www.r-project.org/).

## Results

**Fig. 2:**
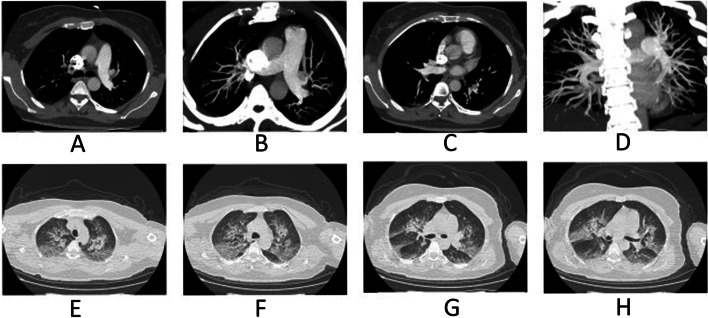
High altitude pulmonary edema (HAPE) in a 39-year-old boy who had dyspnea at rest and worsening cough for 3 days before admission. **A-D** Axial image of CT pulmonary angiogram showing thrombi as filling defects in right main pulmonary artery extending into its branch and in distal left pulmonary artery with extension into its superior branch. **E-H** Multiple lesions of ground glass opacity, patchy lesions and partial consolidation in the CT imaging were presented in the bilateral lung center along the bronchovascular bundle

The occurrence rate of VTE in severe HAPE patients was 38.9% (7/18), including 5 with deep vein thrombosis (DVT) and pulmonary embolism (PE), 2 of whom had DVT only. We followed up the survival rates of all patients within 28 days after a diagnosis of severe HAPE. No patients were lost to follow-up. Moreover, the 18 patients with severe HAPE were no 28-day mortality after patients received anticoagulant therapy (Fig. 2).

Table 1 shows the clinical data for our cohort. The mean age was 43 (34–54) years; 14 (77.8%) patients were men. Comorbidities included respiratory failure, acute high altitude cerebral edema, high altitude polycythemia, pulmonary hypertension. No patient had a history of VTE. Common symptoms at the onset of illness were fever, dry cough, fatigue, dyspnea, diarrhea, headache, Lower extremity swelling and Lower extremity pain. Days from entry into the plateau to onset of illness was 1 (1–2) day. Compared with the non- VTE group, patients with VTE were longer time in hospital (9 (7, 12) vs 13 (11, 19), *P* = 0.027) and a higher proportion of respiratory failure (85.7% [6/7] vs 18.2% [2/11], *P* = 0.013). There were no differences in altitude (permanent residence, birthplace, affected area, difference), symptoms, physical examination, imaging examination and echocardiographic detection (*P* > 0.05 for all) between the VTE and the non- VTE groups.

**Table 1:** Sociodemographic and Clinical Characteristics of 18 patients with High altitude pulmonary edema

Variables	All (*n* = 18)	Non-VTE (*n* = 11)	VTE (*n* = 7)	*P*-value
Age (years)	43 (34, 54)	42 (30, 53)	46 (37, 58)	0.536
Males	14 (77.8)	9 (81.8)	5 (71.4)	1.000
Altitude of permanent residence (m)	2260 (407, 2300)	2260 (280, 2260)	2300 (2260, 2600)	0.069
Altitude of birthplace (m)	2260 (480, 2362)	2260 (450, 2600)	2300 (2260, 2600)	0.126
Altitude of affected area (m)	3700 (3656, 4050)	3700 (3650, 3800)	3700 (3658, 4200)	0.536
Altitude difference (m)	1670 (1397, 2704)	1440 (1390, 3520)	1900 (1400, 1900)	0.659
BMI (kg/m^2^)	23.57 ± 2.04	23.56 ± 2.28	23.57 ± 1.78	0.724
Smoking history	5 (27.8)	5 (45.5)	0 (0.0)	0.101
Drinking history	1 (5.6)	1 (9.1)	0 (0.0)	1.000
Days from entry into the plateau to onset of illness (d)	1 (1, 2)	1 (1, 1)	1 (1, 3)	0.596
Hospitalization days (d)	11 (7, 13)	9 (7, 12)	13 (11, 19)	0.027
History of venous thromboembolism	0 (0.0)	0 (0.0)	0 (0.0)	NA
**Comorbidities**				
Respiratory failure	8 (44.4)	2 (18.2)	6 (85.7)	0.013
Acute HACE	5 (27.8)	2 (18.2)	3 (42.9)	0.326
HAPC	1 (5.6)	0 (0.0)	1 (14.3)	0.389
Pulmonary hypertension	9 (50.0)	4 (36.4)	5 (71.4)	0.335
**Symptoms**				
Fever	8 (55.6)	7 (63.6)	1 (14.3)	0.066
Dry cough	2 (11.1)	1 (9.1)	1 (14.3)	1.000
Dyspnea	17 (94.4)	11 (100.0)	6 (85.7)	0.389
Weakness	15 (83.3)	9 (81.8)	6 (85.7)	1.000
Headache	12 (66.7)	7 (63.6)	5 (71.4)	1.000
Diarrhea	1 (5.6)	0 (0.0)	1 (14.3)	0.389
Lower extremity swelling	1 (5.6)	0 (0.0)	1 (14.3)	0.389
Lower extremity pain	1 (5.6)	0 (0.0)	1 (14.3)	0.389
**Physical examination**				
Respiratory rate (/min)	21 (20, 22)	20 (19, 22)	22 (21, 23)	0.151
Maximum body temperature (°C)	37.8 (37.0, 38.6)	38.0 (37.0, 40.0)	37.4 (37.0, 38.2)	0.536
Heart rate (/min)	101 (69, 112)	100 (72, 108)	102 (61, 126)	0.930
Systolic blood pressure (mmHg)	120 (109, 131)	120 (108, 128)	118 (110, 140)	0.596
Diastolic blood pressure (mmHg)	80 (70, 92)	80 (70, 86)	80 (70, 100)	0.930
**Imaging examination**				
Bronchovascular bundle thickening in both lungs	0 (0.0)	0 (0.0)	0 (0.0)	NA
Diffuse spots in both lungs	7 (38.9)	3 (27.3)	4 (57.1)	0.332
**Echocardiographic detection**				
LA diameter (mm)	33.5 (31.2, 36.0)	33.0 (33.0, 36.0)	34.0 (28.0, 36.0)	0.860
LVESVI (mL/m^2^)	45.0 (42.0, 49.2)	45.0 (42.0, 51.0)	44.0 (42.0, 46.0)	0.425
LVEDVI (mL/m^2^)	28.0 (25.7, 32.2)	28.0 (26.0, 33.0)	28.0 (24.0, 30.0)	0.724
Simpson biplane EF (%)	65.0 (62.7, 67.7)	65.0 (64.0, 70.0)	64.0 (58.0, 67.0)	0.536
RA diameter (mm)	36.0 (31.0, 39.0)	36.0 (31.0, 40.0)	36.0 (31.0, 38.0)	0.425
RV diameter (mm)	23.5 (20.0, 30.2)	23.0 (19.0, 25.0)	26.0 (20.0, 33.0)	0.104
Tricuspid regurgitation	8 (44.4)	3 (27.3)	5 (71.4)	0.145
PA diameter (mm)	23.0 (20.7, 24.2)	22.0 (20.0, 23.0)	24.0 (23.0, 26.0)	0.069

*Abbreviation*: *BMI* Body mass index, *HAPE* High altitude pulmonary edema, *HACE* High altitude cerebral edema, *HAPC* High altitude polycythemia, *LA* Left artery, *LVESVI* Left ventricular end-systolic volume index, *LVEDVI* Left ventricular end-diastolic volume index, *EF* Ejection fraction, *RA* Right artery, *RV* Right ventricle, *PA* Pulmonary artery

Table 2 shows laboratory data and abnormalities at admission, including D-dimer, Activated partial thromboplastin time, Cardiac troponin I and B-type natriuretic peptide. There were no differences in Blood gas analysis, routine blood tests and other blood chemistry tests (*P* > 0.05 for all) between the DVT and the non-DVT groups.

**Table 2:** Laboratory examination of 18 patients with High altitude pulmonary edema at admission

Variables	All (*n* = 18)	Non- VTE (*n* = 11)	VTE (*n* = 7)	*P*-value
**Blood gas analysis**				
PH	7.46 ± 0.05	7.45 ± 0.05	7.48 ± 0.05	0.479
Lactic acid (mmol/L)	1.2 (1.0, 1.8)	1.2 (0.8, 1.5)	1.8 (1.1, 2.1)	0.179
Oxygenation index (mmHg)	202 (168, 253)	229 (190, 254)	178 (141, 253)	0.246
PO_2_ (mmHg)	61.1 (53.4, 76.0)	63.4 (55.0, 75.4)	58.8 (46.4, 83.4)	0.536
PCO_2_ (mmHg)	33.0 (28.5, 36.7)	32.0 (28.4, 35.0)	36.0 (28.5, 39.2)	0.328
**Routine blood tests**				
WBC (×10^9^/L)	8.72 (5.39, 13.64)	9.13 (5.05, 13.25)	8.10 (5.51, 15.53)	1.000
LY (×10^9^/L)	1.26 (0.95, 1.65)	1.29 (1.04, 1.80)	1.25 (0.69, 1.33)	0.724
NE (×10^9^/L)	7.35 (4.17, 11.06)	7.67 (4.40, 10.71)	7.04 (3.48, 13.21)	1.000
NE/LY	6.93 (3.01, 9.99)	7.06 (3.10, 9.36)	6.80 (2.74, 10.17)	1.000
PLT (×10^9^/L)	157 (119, 191)	169 (140, 226)	136 (100, 163)	0.085
Hemoglobin (g/L)	162 (148, 176)	163 (149, 170)	161 (145, 184)	0.724
**Blood chemistry tests**				
CRP (mg/L)	1.98 (0.52, 4.75)	4.01 (0.66, 6.01)	0.81 (0.40, 2.99)	0.230
Procalcitonin (ng/mL)	0.06 (0.02, 0.14)	0.02 (0.02, 0.09)	0.10 (0.06, 0.22)	0.069
D-dimer (μg/mL)	3.23 (2.22, 7.64)	2.90 (1.75, 3.37)	7.81 (4.62, 9.60)	0.003
PT (sec)	12.05 (10.88, 13.30)	11.80 (10.60, 12.70)	12.30 (11.10, 14.40)	0.375
APTT (sec)	25.7 (21.6, 28.1)	26.30 (24.80, 30.10)	21.50 (19.00, 27.50)	0.044
Total protein (g/L)	60.5 (51.4, 66.0)	62.6 (53.2, 67.4)	58.8 (51.0, 60.7)	0.285
Albumin (g/L)	36.1 (29.9, 41.4)	36.3 (32.5, 42.1)	33.0 (28.0, 40.3)	0.425
AST (U/L)	23 (19, 33)	21 (18, 30)	30 (22, 42)	0.330
ALT (U/L)	32 (21, 52)	30 (22, 43)	36 (17, 75)	0.596
LDH (IU/L)	244 (238, 343)	243 (238, 372)	276 (233, 343)	0.740
Missing	1 (5.6)	1 (9.1)	0 (0.0)	
BUN (mmol/L)	5.68 (4.66, 8.59)	5.18 (4.00, 6.87)	7.84 (5.25, 8.62)	0.328
Creatinine (μmol/L)	78.6 (68.2, 91.2)	74.0 (71.0, 89.6)	84.0 (60.0, 92.0)	0.536
cTnI (pg/mL)	15.17 (0.79, 92.76)	1.61 (0.40, 22.12)	256.13 (17.60, 504.34)	0.004
CKMB (U/L)	16.6 (13.7, 21.2)	19.3 (14.0, 24.0)	15.1 (11.8, 21.0)	0.285
BNP (pg/mL)	33.5 (31.2, 36.0)	28.0 (24.0, 35.0)	52.0 (41.8, 400.0)	0.006

*Abbreviation*: *PO*_*2*_ Partial pressure of oxygen, *PCO*_*2*_ Partial pressure of carbon dioxide, *WBC* White blood count, *LY* Lymphocyte count, *NE* Neutrophil count, *NE/LY* Ratio of neutrophil count to lymphocyte count, *PLT* Platelet count, *CRP* C-reactive protein, *PT* Prothrombin time, *APTT* Activated partial thromboplastin time, *AST* Aspartate aminotransferase, *ALT* Alanine transaminase, *LDH* Lactate dehydrogenase, *BUN* Blood urea nitrogen, *cTnI* Cardiac troponin I, *CKMB* Creatine kinase-MB, *BNP* B-type natriuretic peptide

Treatments of patients with high altitude pulmonary edema are shown in Table 3. Of the 18 patients, 18 (100%) all received oxygen therapy, noninvasive mechanical ventilation and diuretics therapy, 4 (36.4%) received aminophylline therapy, 1 (5.6%) received vasodilator drugs therapy, 7 (38.9%) patients were given anticoagulant therapy, and 3 (16.7%) patients received Antiplatelet therapy before CTPA, cardiac ultrasound or venous ultrasound scanning for VTE. Most patients (10/18 [55.6%]) received hormone therapy, 4 (22.2%) with sedative treatment and 5 (27.8%) with gamma globulin. Compared with the non-VTE group, patients with VTE have similar proportion of VTE prophylaxis (0 [0.0%] versus 2 [18.2%]; *P* = 0.497). There was no statistically significant difference in the other treatments between the VTE and non-VTE groups (*P* > 0.05 for all).

**Table 3:** Treatments of 18 patients with High altitude pulmonary edema

Variables	All (*n* = 18)	Non- VTE (*n* = 11)	VTE (*n* = 7)	*P*-value
Aminophylline	4 (36.4)	4 (36.4)	0 (0.0)	0.119
Vasodilator drugs	1 (5.6)	1 (9.1)	0 (0.0)	1.000
Venous thromboembolism prophylaxis	2 (11.1)	2(18.2)	0(0.0)	0.497
Antiplatelet therapy	3 (16.7)	1 (9.1)	2 (28.6)	0.528
Sedative treatment	4 (22.2)	1 (9.1)	3 (42.9)	0.245
Hormone	10 (55.6)	6 (54.5)	4 (57.1)	1.000
Gamma globulin	5 (27.8)	2 (18.2)	3 (42.9)	0.326

## Discussion

This study explored the prevalence and clinical characteristics of venous thromboembolism from severe high altitude pulmonary edema in plateau regions. We performed a single-institution retrospective study of patients with confirmed severe HAPE and found a high prevalence of VTE and an association between VTE and longer time in hospital, respiratory failure, the shortened APTT and the higher level of D-dimer in hospitalized patients with severe HAPE. In our current study of the largest sample size to date, little is known about the occurrence rate and the association of VTE in severe HAPE.

We observed that the occurrence rate of VTE in our study population was 38.9% (7/18 studied), 26.9% (7/26 of all severe HAPE studied) and 2.65% (7/264 of all patients in our center). This prevalence appears to be higher than that reported in the literature [7–10], which reported that the rates of objectively confirmed VTE in 4 prospective studies ranged from 13 to 31% and suggested a potential role for thromboprophylaxis in patients requiring critical care. This rate was also higher than that reported for many other hospitalized patients [11, 12] and series of patients in ICUs reported from China [13].

Several reasons probably account for the high prevalence of DVT in severe HAPE patients. First, most of the previously mentioned studies focused on critically ill patients who were in the ICU for different diseases. HAPE is an abnormally high pulmonary artery pressures due to multiple factors. Most studies suggest that HAPE is associated with damaged endothelium and alveolar epithelium caused by inflammatory responses under hypoxic conditions, which involve several pathways and mediators, including hypoxia-inducible factor, vascular endothelial growth factor, endothelin-1, and inducible nitric oxide synthase, and also involving sodium channels that regulate water transport, Na-K-ATPase, and aquaporin, pulmonary hemodynamic changes and higher hydrostatic pressure play a vital role in the acute and rapid progression of HAPE [14–16]. Second, there are aspects of altitude excursions that increase blood viscosity, for example, dehydration causing hemoconcentration, and polycythemia, in addition to the compensatory rise in hematocrit with acclimatization, which are likely to increase VTE risk. Furthermore, Studies have shown hypoxia and low temperature at high altitude can induce hypercoagulabilitys to increase thromboembolic events at high-altitude [17].

Outcome analyses clinical characteristics of venous thromboembolism onset from severe high altitude pulmonary edema in plateau regions, including longer time in hospital, respiratory failure, the shortened APTT and the higher level of D-dimer. Therefore, we speculate that the inflammatory state may promote venous thrombosis under hypoxic conditions. Coagulation activation could also be associated with a sustained infammatory response [18]. D-dimer is an important indicator for diagnosing of patients with VTE, and its increase is important significance for the differential diagnosis of patients with symptomatic VTE. Le Roux et al. demonstrated that the levels of D-dimer levels increased significantly at 6542 m after 1 week and at 3 weeks compared to those observed at sea level in seven climbers [19]. Analogously, Pichler Hefti et al. found that D-dimer levels increased with increasing altitude in Muztagh Ata, China [20]. Our data showed that serum D-dimer levels in patients with VTE in severe high altitude pulmonary edema were higher than non-VTE groups. This suggests that D-dimer, as an important differential index for VTE diagnosis, still has diagnostic efficacy in plateau regions. A transcriptomic and proteomic analysis of platelets demonstrated that plateau regions were associated with the upregulation of proteins with thrombosis and platelet activation without thrombosis in plateau regions -residing patients compared to subjects residing at low-altitude [21]. Moreover, a novel genome-wide expression analysis performed by Jha et al. showed that genes associated with the coagulation cascade and platelet activation were significantly upregulated in patients with VTE at plateau regions [22]. These studies indicate that platelet activation may induce thrombosis.

Prophylaxis for VTE [23, 24] and for extended-duration VTE [25, 26] has been investigated in clinical trials to improve clinical outcomes in severely or critically ill patients. Regrettably, the proportion of thromboprophylaxis is too low in our cohort which 2 of 18 (11.1%) patients were given VTE prophylaxis. There was no statistically significant difference between the VTE and non-VTE groups (0 [0.0%] versus 2 [18.2%]; *P* = 0.497). Our data suggest that there is a possible protective effect of prophylaxis for VTE in the higher risk in this cohort. This also suggests that for HAPE, thromboprophylaxis strategies should fully be recognized and strengthened, including moderately increasing the dose of anticoagulant drugs. Because our sample size is limited, the effect of VTE prophylaxis on hospitalized patients with COVID-19 warrants further investigation.

Although these findings are not surprising, given that our patient population represented severely ill patients at high risk for VTE, our data raised the question of screening for VTE, risk stratification, and potential VTE prophylaxis to improve outcomes in hospitalized patients with HAPE. Meanwhile, since VTE has no specific clinical manifestations, it can be easily diagnosed as other respiratory diseases at the initial stage, and a combination of these diseases cannot be ruled out. These results make it difficult to diagnose VTE. Therefore, more attention should be paid to the high-risk VTE groups in HAPE groups and VTE prophylaxis.

This study has some limitations. First, this is a single center retrospective study, as the sample size is small, and it is difficult to exclude accidental errors. Second, due to the critical condition of patients with severe HAPE, CTPA examinations were restricted, which significantly underestimated the prevalence of VTE. Thus, Prospective multi-center large sample studies might be needed in the future to further confirm the findings in our current study.

## Conclusions

The incidence of VTE is extremely high in patients with severe HAPE and may be associated with adverse outcomes. The clinical characteristics for VTE are longer time in hospital, respiratory failure, the shortened APTT and the higher level of D-dimer in severe HAPE. We suspect that VTE is probably an additional risk factor for the death of severe HAPE in hospitalized patients. The analysis of severe HAPE may help to provide more accurate screening for VTE and lead to corresponding measures to improve the clinical outcome of patients with severe HAPE.

## Data Availability

All data analyzed during the study are presented in the main manuscript. The anonymous dataset is available from the corresponding author upon reasonable request.

## Abbreviations

VTE: Venous thromboembolism
HAPE: High altitude pulmonary edema
CTPA: Computed tomographic pulmonary angiography
DVT: Deep vein thrombosis
PE: Pulmonary embolism
APTT: Activated partial thromboplastin time
BMI: Body mass index
ICU: Intensive care unit
